# 

**Published:** 1898-12

**Authors:** 


### EDITORIAL.

### The National Dental Technic Association

Meets in this city on December 28th and 29th, at the Grand Hotel. This is an association of active, energetic teachers from various dental colleges of our country. The organization has been in existence about six years and has accomplished a grand work thus far, and promises still more for the future. We trust that every reputable college of the country will be represented at this meeting, and that the various members will come prepared to make the occasion one of interest and great profit.

### PRELIMINARY PROGRAM OF THE SIXTH ANNUAL MEETING OF THE NATIONAL SCHOOL OF DENTAL TECHNICS.

CINCINNATI, OHIO, December 28-29, 1898.

Ten A. M. (1) Organization and reports of Executive Board; (2) President's Address, G. V. Black, D.D.S., M.D.; (3) Discussion opened by Drs. Guilford, Brophy, and H. A. Smith. 2 P. M. (1) "The Value of and Necessity for a Uniform Graded Course," by G. V. I. Brown; (2) Discussion by Drs. M. T. McLean, E. C. Kirk, J. Taft. 8 P. M. (1) Report of Committee on Syllabus for Prosthetic Technics, by N. S. Hoff; (2) Discussion by Drs. G. Molyneaux, J. H. Lewis, J. G. Harper, H. B. Tileston; Master of Exhibits, Dr. Grant Molyneaux; (3) Report of Committee on Syllabus for Operative Technics, by T. E. Weeks; (4) Discussion by Drs. J. A. Dale, J. F. Stephan, W. G. Foster, L. S. Tenny. 10 A. M. Symposium of Teaching Methods. (1) Conduct of the Operative Clinic, Dr. W. H. Whitslar; (2) Text-Books and Recitation Methods, Dr. C. M. Wright; (3) Lecture and Class Methods, Dr. H. H. Burchard; (4) General Discussion. 2

P. M. (1) Steel Technics, Dr. George E. Snow; (2) Discussion by Drs. G. H. Wilson, J. D. Patterson, G. V. Black. 4 P. M. (1) Teaching Cavity Preparation, Dr. C. N. Johnson; (2) Discussion by Drs. W. E. Harper, J. H. Daly, W. J. Brady. 8 P. M. (1) Voluntary Papers; (2) Election of Officers; (3) Adjournment. D. M. CATTELL, *Sec'y-Treas.*

### Close of the Year.

This number of THE REGISTER closes the fifty-second year of its existence. It has passed through its career thus far without a single break in the regularity of its publication.

THE REGISTER is the second oldest dental journal in the world that has had continuous publication. The *American Journal of Dental Science* was first published in 1839, the DENTAL REGISTER in 1847. Between these two periods six dental journals were issued, three in 1843, one in 1844, one in 1845, one in 1846. These, however, were short-lived. The *New York Dental Recorder* had the longest career, namely from 1846 to 1856, and was edited by C. C. Allen, and the last volume by C. A. Ballard, of New York.

The *Quarterly Journal of Dental Science* of 1843 was edited by James Robinson, of London, the *Dental Forceps* of 1844 was also published in London. These ceased publication after a year or so.

For the first nine years the DENTAL REGISTER was published by Dr. James Taylor, of Cincinnati, and from Volume X. to Volume XXV. it was issued under the joint editorship of J. Taft and George Watt. It was published as a quarterly up to June, 1859, then it was made a monthly, and has so continued from that time to the present. It originally bore the title of the DENTAL REGISTER OF THE WEST, but in 1865 the latter part of the title was dropped. In 1873 and from that time to the present it has been edited and owned by J. Taft. THE REGISTER aims to give such matter as will be permanently valuable to the profession, both to the student and the practitioner.

### CONTENTS.

COMMUNICATIONS.

Report of the Committee on Foreign Relations of the National Association of Dental Faculties........................................ 551

PROCEEDINGS.

Tri-State Meeting........................................................ 566
"Degeneration and Decay of Oral Tissues from Lack of Exercise," 574

EDITORIAL.

The National Dental Technic Association.................................. 591

Close of the Year........................................................ 592

### THE DENTAL REGISTER,

A MONTHLY MAGAZINE DEVOTED TO THE INTERESTS OF THE

**DENTAL PROFESSION.**

*Terms Reduced to* $2.00 *Per Year. Single Copies,* 25 *Cents.*

**EDITED BY PROF. J. TAFT, D.D.S.**

—AND—

**Published by SAM'L A. CROCKER & CO., Ohio Dental and Surgical Depot.**

The volume commences in January and closes in December.

The REGISTER being issued monthly is always fresh, therefore Indispensable to the practitioner who desires to keep posted up with the new inventions and improvements which are daily developed. Neither expense nor effort will be spared to give value and variety to its contents. Articles will be illustrated with well executed engravings whenever they will add to the interest or assist to explanation.

Its extensive circulation and frequency of issue make it one of the BEST DENTAL ADVERTISING MEDIUMS in the United States.

All subscriptions must begin with January or July.

Local or special notices, five lines or less, will be inserted for $3 each insertion; over five lines, 50 cts. per line.

All advertising bills payable in advance, or at furthest, after the issue of the first number containing the advertisement. All transient advertisements to be paid for in advance.

☞CONTRIBUTIONS SOLICITED.

Exclusively Editorial Communications should be addressed to the Editor.

The latest number of the REGISTER may be ordered with or without other goods at any time, and all letters should be addressed to [illegible]

